# Current Insights into Combination Therapies with MAPK Inhibitors and Immune Checkpoint Blockade

**DOI:** 10.3390/ijms21072531

**Published:** 2020-04-05

**Authors:** Min Hwa Shin, Jiyoung Kim, Siyoung A. Lim, Jeongsoo Kim, Kyung-Mi Lee

**Affiliations:** Department of Biochemistry and Molecular Biology, College of Medicine, Korea University, Seoul 02841, Korea

**Keywords:** combination therapy, MAPK targeted therapy, BRAF inhibitor, MEK inhibitor, immune checkpoint inhibitor, PD-1, PD-L1, CTLA-4

## Abstract

The recent development of high-throughput genomics has revolutionized personalized medicine by identifying key pathways and molecular targets controlling tumor progression and survival. Mitogen-activated protein kinase (MAPK) pathways are examples of such targets, and inhibitors against these pathways have shown promising clinical responses in patients with melanoma, non-small-cell lung cancer, colorectal cancer, pancreatic cancer, and thyroid cancer. Although MAPK pathway-targeted therapies have resulted in significant clinical responses in a large proportion of cancer patients, the rate of tumor recurrence is high due to the development of resistance. Conversely, immunotherapies have shown limited clinical responses, but have led to durable tumor regression in patients, and complete responses. Recent evidence indicates that MAPK-targeted therapies may synergize with immune cells, thus providing rationale for the development of combination therapies. Here, we review the current status of ongoing clinical trials investigating MAPK pathway inhibitors, such as BRAF and MAPK/ERK kinase (MEK) inhibitors, in combination with checkpoint inhibitors targeting programmed death protein 1 (PD-1), programmed death-ligand 1 (PD-L1), and cytotoxic T cell associated antigen-4 (CTLA-4). A better understanding of an individual drug’s mechanism of action, patterns of acquired resistance, and the influence on immune cells will be critical for the development of novel combination therapies.

## 1. Introduction

In recent years, the focus of cancer therapeutics has shifted from the treatment of cancers based on type and histology to those targeting specific gene mutation and its dysregulation. Accordingly, cancer treatment regimens have advanced considerably with the development of specific inhibitors aimed at oncogenic and mutated proteins [1]. Table 1 summarizes the tumor-driver mutations identified in melanoma, non-small-cell lung cancer (NSCLC), colorectal cancer (CRC), pancreatic cancer, and thyroid cancer [2].

Due to the genetic heterogeneity of cancer, standard treatments, including chemotherapy and radiation, are effective only in a subset of patients diagnosed early and with tumors that have low invasive properties. This inherent heterogeneity of cancer lends itself to the growing field of precision and personalized medicine. Personalized therapies could inhibit cancer growth and at the same time increase antitumor immunity in patients more efficiently. Targeted therapies and immunotherapies are two approaches that can generate individualized treatment. Targeted therapies target various oncogenic proteins that contribute to cancer development. Although these targeted therapies can lead to a substantial reduction of tumor size in various cancers, clinical responses tend to be temporary, and often result in tumor escape and relapse after initial treatment. Table 2 summarizes targeted therapies approved by the United States (US) Food and Drug Administration (FDA) for the treatment of cancer [2]. 

Compared to targeted therapy, immunotherapy has demonstrated more enduring responses in patients with multiple types of cancer. Notably, checkpoint blockade, one form of immunotherapy, has been shown to lead to long-lasting responses; however, the response rate is lower than that of targeted therapies. Checkpoint blockade therapies target checkpoint proteins, which are involved in suppressing the immune system. Table 3 summarizes FDA-approved monoclonal antibodies (mAbs) and their targets for checkpoint blockade, as well as other antitumor immunotherapies [2].

Despite the benefits of targeted therapies and immunotherapies, they are also associated with a number of limitations. Such limitations may be overcome by combining a targeted therapy and an immunotherapy [3], and several clinical trials investigating combination therapy are ongoing to assess the safety and efficacy of these approaches. Here, we review the current status of therapies targeting the MAPK pathway and immunotherapies, mainly focusing on melanoma and NSCLC, as well as other solid cancers, such as pancreatic cancer, CRC, and thyroid cancer (clinicaltrials.gov). Major drivers of these cancers include BRAF (V600E, V599E) and/or KRAS (G12C, G12D) along the MAPK pathways while immunotherapies were considered as a monotherapy or in combination in those tumors expressing PD-1 ligands. Melanoma and NSCLC were chosen since they were shown to be refractory to conventional treatment but exhibited long-lasting responses to immunotherapies. Furthermore, colorectal cancer patients with microsatellite instability were shown to benefit from anti-PD-1 therapy while some of these patients also express BRAF V600E [4]. In addition to these cancers, drivers of pancreatic cancers and thyroid cancers have been shown to exhibit KRAS (G12C, G12D) and BRAF (V599E) among MAPK pathways [5]. Therefore, we expect that combination of MAPK-targeted therapies with immune checkpoint blockades could potentially compensate and synergize each other to produce long lasting antitumor effects. 

## 2. Therapies Targeting the MAPK Pathway

**Figure 1:**
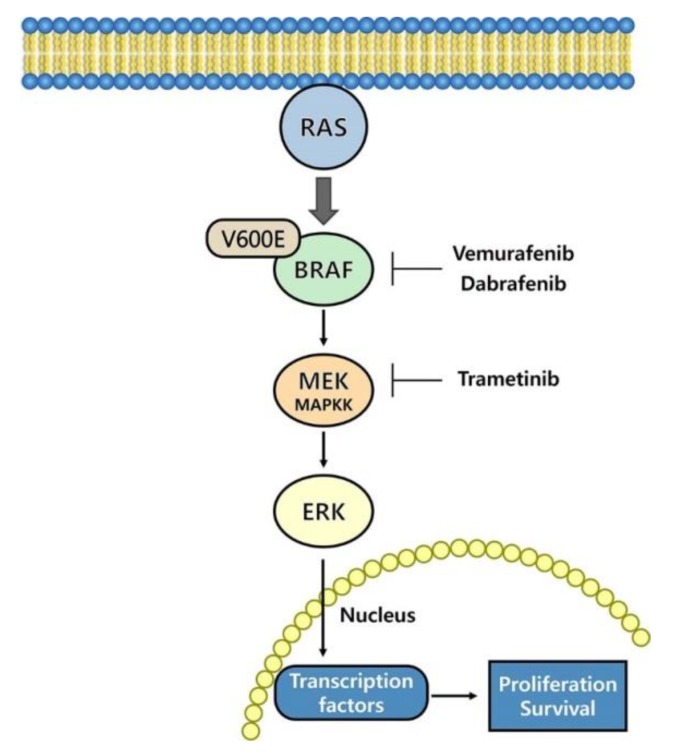
Mitogen-activated protein kinase (MAPK) pathways and BRAF and MAPK/ERK kinase (MEK) inhibitors.

Major advances have been made in the treatment of cancer through genetic and genomic tools to investigate the mechanisms of progression of several types of cancer. Targeting the MAPK pathway is a therapeutic approach aimed at oncogenic and mutated/dysregulated proteins. The main targets of these treatments have been the MEK (MAPK/ERK kinase) and BRAF proteins in the MAPK pathway. A schematic illustration of the MAPK signaling pathway and FDA-approved inhibitors of this pathway is shown in Figure 1. 

In melanoma, an activating point mutation at V600 in BRAF is the main oncogenic driver [6,7]. This BRAF mutation activates signaling of the MAPK pathway, and promotes cancer cell proliferation and immune escape [8,9].

Subsequently, BRAF inhibitors were developed for the treatment of patients with melanoma. However, one of the early BRAF inhibitors, sorafenib, demonstrated toxicity, including non-specific side effects, and resulted in low clinical responses in patients with melanoma [10]. A more effective inhibitor (vemurafenib) was later developed targeting the V600-mutated site of BRAF; this demonstrated favorable responses and resulted in an increased rate of overall survival (OS) in multiple clinical trials [11]. Dabrafenib, which also targets the BRAF V600 mutation in melanoma, has demonstrated improved OS in clinical trials [12]. Vemurafenib and dabrafenib were approved by the FDA as monotherapies in 2011 and 2013, respectively. Initial in vitro and in vivo studies using BRAF-targeted therapies demonstrated increased melanoma antigen expression with tumor-infiltrating CD8+ T cells, and decreased levels of VEGF and immunosuppressive cytokines. These findings demonstrate heightened CD8+ T cell targeting to tumors as well as increased levels of granzyme B and perforin—two proteases that control T cell-induced tumor apoptosis [12]. 

For patients without the BRAF mutation, an inhibitor of MEK—a kinase downstream of RAF in the MAPK-signaling pathway—such as trametinib (FDA approved in 2013), demonstrated favorable clinical responses [13]. MEK inhibitors can induce melanocyte-inducing transcription factor (MITF) and melanocyte-derived antigen expression, and enhance T cell infiltration to tumors similar to BRAF inhibitors. MEK inhibitors were also shown to generate an antitumor immune response by hindering the interaction between tumor cells and M2-like macrophages, thus allowing the tumor-specific effector T-cells to be trafficked to tumors [14]. 

A major issue with both BRAF and MEK inhibitors is the development of gene resistance to the drugs. To reduce this resistance, combination therapy with a BRAF and a MEK inhibitor has been developed as a therapeutic approach for melanoma patients with the BRAF mutation. This combination aims to overcome resistance to a single inhibitor by focusing on vertical elements along the MAPK pathway. Combined treatment with BRAF and MEK inhibitors is currently approved, but only small advances in progression-free survival (PFS) and OS compared with BRAF inhibitor monotherapy have been reported. However, resistance continues to be an issue [15]. 

Inhibition of the MAPK pathway in both BRAF-mutant and wild-type melanoma cells resulted in increased tumor antigen expression and T-cell function [7]. Patients with melanoma treated with a BRAF inhibitor have also demonstrated enhanced antigen expression in tumors [16]. The immune effects of MAPK pathway inhibitors are reported to affect T cells and dendritic cells, regardless of the BRAF mutation status [17]. Patients treated with BRAF inhibitors have demonstrated preserved T cell activity and viability, highlighting their potential combination with immunotherapy [18]. However, the immune response to BRAF-targeted therapies occurs early, but this response is lost upon tumor progression [19]. Furthermore, PD-1 and Tim-3 expression were found to be increased in T cells and in their corresponding ligands in tumors [20]. These phenotypes support the rationale for combination therapy with a MAPK-targeted inhibitor and immune checkpoint blockade (anti-PD-1, anti-PDL-1, or Tim-3). 

## 3. Immune Checkpoint Blockade

Cancer immunotherapy aims to improve the ability of the patients’ immune system to destroy tumor cells. The first immunotherapy regimen developed used interleukin 2 (IL-2) to treat patients with metastatic melanoma [21]. IL-2 has been reported to increase T-cell activation in a non-specific manner [9]. However, due to the serious toxicity and low response rates, high doses of IL-2 are now rarely used as monotherapy [22]; however, low-dose IL-2 in combination with other therapies, such as adoptive cell transfer, have demonstrated therapeutic efficacy [23]. 

**Figure 2:**
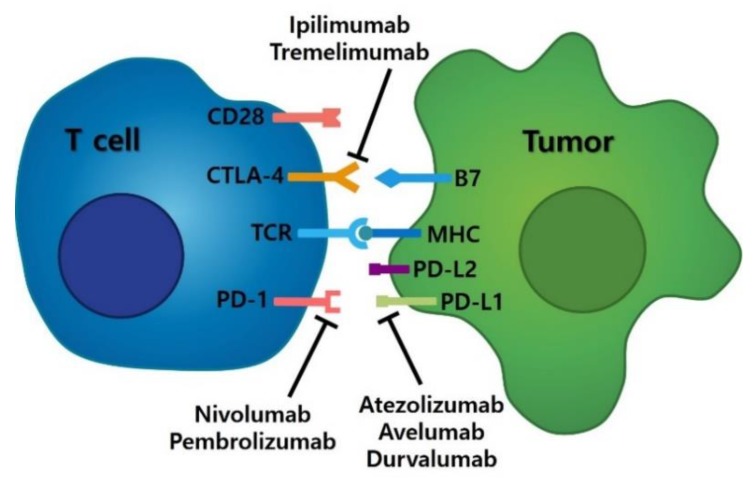
Checkpoint inhibition with PD-1, PD-L1, and CTLA-4 blockade.

Immune checkpoints represent an area of cancer immunotherapies for which treatments are being developed. The major function of immune checkpoints is to limit immune cell activation to maintain immune homeostasis while inhibiting the development of autoimmunity. Immune checkpoint molecules are upregulated by tumor cells within the tumor microenvironment to repress the anti-cancer response of the immune system. Therefore, the use of specific mAbs to block immune checkpoints is expected to reverse the suppression of tumor-specific immune cells, including T cells and natural killer (NK) cells [24,25]. Monoclonal antibodies against immune checkpoint receptors, such as CTLA-4, PD-1, and PD-L1, have demonstrated significant results in the treatment of multiple advanced cancers, including melanoma, NSCLC, head and neck, and bladder cancers [26]. The interactions of PD-1, PD-L1, and CTLA-4 with their cognate ligands and clinical trials with various inhibitors are illustrated in Figure 2 and Table 4, respectively.

Ipilimumab, a monoclonal antibody against CTLA-4, was the first inhibitor developed for the treatment of patients with stage IV melanoma [27]. CLTA-4 prevents early T-cell activation; it is not present on resting T cells but is upregulated immediately upon T cell activation [9]. CTLA-4 competes with CD28 for their shared ligands, B7-1 and B7-2 (also known as CD80 and CD28, respectively), resulting in the negative regulation of T-cell signaling [28]. PD-1 is another immune checkpoint molecule. Similar to CTLA-4, PD-1 is generally upregulated on activated T cells [29], and negatively regulates T-cell receptor signaling and tumor killing functions by interacting with PD-L1 and PD-L2 [30]. Crosslinking of PD-1 also increases the apoptosis of cytotoxic T cells [31]. PD-1 blockade (for example, with nivolumab) has been investigated in multiple clinical trials in patients with melanoma, NSCLC, and CRC [32]. Patients with advanced melanoma enrolled in trials investigating PD-1 therapies have demonstrated a response rate of 28%, with durable results lasting more than 1 year in 50% of responding patients [33].

When used as monotherapy, currently approved checkpoint inhibitors do not lead to durable clinical responses in almost 80% of cancer patients. Patients who responded well to checkpoint therapy often had a significant number of tumor-infiltrating T cells before starting treatment. Thus, a limitation of checkpoint therapy is the lack of available activated T cells that can respond to the therapy. In addition, cancers that demonstrate an initial response may become resistant to checkpoint therapy, which would further reduce treatment efficacy [34].

Among anti-CTLA-4 and PD-1 treatments, the latter have been shown to be more effective. A study comparing ipilimumab with pembrolizumab (another PD-1 antibody) reported 6-month PFS of 26.5% and 47.3%, respectively. Anti-PD-1 therapies have also been shown to result in fewer adverse events compared with anti-CTLA-4 therapies. This is likely due to the expression of PD-1 on mature T cells (such as dendritic cells and macrophages), while CLTA-4 is present on T cells throughout the body, including those in the lymph nodes and skin [35].

Combination therapy using PD-1 and CLTA-4 antibodies has been studied to enhance efficacy and reduce toxicity. Since CTLA-4 and PD-1 therapies function through different mechanisms, their use in combination can generate a synergistic effect, thus limiting the side effects from a single checkpoint inhibitor. Checkpoint monotherapy also has been shown to activate alternative T-cell checkpoints; thus, therapy targeting one checkpoint will result in the upregulation of another checkpoint, which may exacerbate tumor effects [35]. Recent clinical trials showed that blocking CTLA-4 and PD-1 simultaneously in patients with advanced melanoma strengthened the function of tumor-specific T cells, but resulted in off-target/on-target side effects [36]. Consequently, alternative combinations with other checkpoint inhibitors or targeted therapies are being developed in an attempt to reduce toxicities. 

## 4. Combination of Immunotherapy and Targeted Therapy

Melanomas, like many types of cancer, are capable of evading cell death by suppressing the immune system. One of these mechanisms involves the activation of PD-L1 expression by the release of cytokines (such as interferon-gamma) in tumor-infiltrating lymphocytes (TILs). Immune resistance has been demonstrated in BRAF inhibitor-resistant melanoma cell lines with increased PD-L1 expression, which permitted host immune cell evasion [15]. Despite the development of resistance, in the early stages of treatment, BRAF (and MAPK) inhibitors can stimulate the immune response against tumors.

BRAF and MEK inhibitors can also enhance intratumoral T cell infiltration. Many studies have shown increased T cells in BRAF-mutated melanomas following treatment with MAPK pathway inhibitors; however, this increase is lost as therapy progresses. MAPK pathway inhibitors can also protect CD8 effector T cells from death through chronic T-cell receptor stimulation. As noted above, MEK inhibitors exert their immune-stimulatory effects by increasing melanocyte-derived antigen expression, increasing T cell infiltration, and reducing the interaction between tumor cells and M2-like macrophages. This increase in tumor antigen levels may augment antitumor T-cell responses [15]. However, MEK inhibitors may have adverse effects on naive T cell proliferation, viability, and interferon gamma secretion. 

While MAPK inhibitors can induce temporary responses, many of these are not durable. Melanoma patients carrying a BRAF mutation have been shown to exhibit some short-term benefits from targeted therapies, such as MAPK inhibitors. Conversely, immunotherapy (such as immune checkpoint blockades) has been shown to induce longer-term responses in approximately one-third of patients [14]. Recent studies suggested that short-term inhibition of both BRAF and MEK in combination with anti-PD-1/L1 antibodies could enhance tumor immune infiltration, and improve tumor control in a CD8 T cells-dependent manner. Combination therapy with MAPK inhibitors and immune checkpoint inhibitors may overcome some of the limitations associated with the monotherapies.

Clinical trials are currently ongoing to investigate combinations of immune checkpoint blockades (particularly anti-PD-1/L1 therapies) with BRAF and/or MEK inhibitors. A long-term study is needed to determine the potential toxicity of each single agent when used in combination therapy. One phase I study reported preliminary clinical activity and a tolerable safety profile for an anti-PD-L1 antibody in combination with dabrafenib (BRAF inhibitor) and trametinib (MEK inhibitor) in BRAF-mutated melanoma as well as in BRAF wild-type melanoma [37]. An ongoing phase II study is currently investigating the efficacy of pembrolizumab (anti-PD-1 therapy) in combination with intermittent or continuous administration of BRAF and MEK inhibitors (dabrafenib and trametinib) in patients with advanced melanoma (NCT02625337) [38,39].

Optimizing the sequence in which checkpoint inhibitors and targeted therapies are given (sequential rather than in combination) may also have potential benefits, such as reduced toxicity and reduced costs associated with concurrent approaches used over long periods. In a phase II trial (CA184-240/NCT01673854), ipilimumab was introduced after 6 weeks of vemurafenib therapy, preventing the liver toxicity observed when the treatments were used simultaneously [38]. In that trial, sequential treatment was tolerable, and resulted in a median PFS of 4.4 months and an overall response rate (ORR) of 30% following initial vemurafenib and ipilimumab treatment [38]. Regardless of whether BRAF and/or MAPK inhibitors are administered sequentially or in combination with an immune checkpoint inhibitor, treatment with both agents leads to more durable responses in patients with BRAF-mutated melanoma [38]. This is likely due to the compensatory and synergistic relationship between the two types of therapies. Approaches to integrate targeted therapies with immunotherapies have helped to overcome the limitations of each individual regimen and strengthen the responses to monotherapy. Here, we review ongoing clinical trials in patients with melanoma, NSCLC, colon cancer, pancreatic cancer, and thyroid cancer. We also added adverse effects of the combination therapies of melanoma. Since clinical trials of other types of cancer have not been completed or terminated, their adverse side effects have not been available yet.

### 4.1. Melanoma

The primary treatment option for patients with early-stage melanoma is surgery; however, surgery is not the best solution for patients with advanced melanoma due to the high rate of metastasis. Generally, conventional chemotherapy is used for patients with late-stage melanoma, but the response rate is very poor, at approximately 5% [40,41]. However, following the recent development of targeted therapies and immunotherapies, substantial improvements have been made in the prognosis of melanoma. The BRAF V600 mutation leads to the constitutive activation of the MAPK pathway in more than 50% of patients with melanoma [42]. Following approval of the BRAF inhibitor vemurafenib by the FDA in 2011 for patients with advanced melanoma, PFS and OS rates have improved significantly [11]. In 2013, another BRAF inhibitor, dabrafenib, was approved by the FDA, and was shown to improve function compared with vemurafenib [43]. These inhibitors were found to result in remarkable clinical responses in the short term; however, long-term responses are uncommon due to acquired resistance [44].

Since MAPK pathways are reactivated by the BRAF mutation, MEK (MAPK Kinase) inhibitors, including trametinib, have been developed. Trametinib inhibits MEK1/2 and was the first MEK inhibitor to be approved by the FDA for the treatment of patients with advanced melanoma [45]. The combination of encorafenib (a BRAF inhibitor) and binimetinib (a MEK inhibitor) was recently approved by the FDA for the treatment of patients with advanced melanoma. Patients with BRAF-mutated melanoma have shown improved PFS and OS following combination treatment with these MAPK pathway inhibitors [46]; however, decreased clinical responses have been reported in patients following several months of treatment [47]. Ongoing clinical trials investigating the treatment of melanoma are shown in Table 5 [38]. 

To enhance the clinical response to therapies targeting the MAPK pathway, clinical trials are investigating regimens that incorporate both targeted therapies and immunotherapies, specifically immune checkpoint inhibitors. For example, NCT01400451 is investigating the efficacy and safety of vemurafenib (BRAF inhibitor) in combination with ipilimumab (CTLA-4-Ab) for the treatment of patients with metastatic melanoma expressing an activated mutant form of BRAF V600E [38]. NCT01400451 was closed to enrollment and Phase II was not initiated, because the drug combination was not fully tolerated, and the highest tolerable dosage was not established [38]. The adverse event was unexpected grade 2/3 hepatotoxicity [38]. NCT01673854 is investigating the safety of vemurafenib, administered at 960 mg for 6 weeks along with ipilimumab in a sequential manner, to BRAF V600-mutated melanoma patients [38]. No severe hepatotoxicity was observed, but a grade 3/4 skin adverse event has been reported [38]. NCT03554083 is studying the effect of combined treatment with cobimetinib (MEK inhibitor) and atezolizumab (PD-L1-Ab), and with vemurafenib (BRAF inhibitor), cobimetinib, and atezolizumab for the treatment of patients with high-risk stage III melanoma [38]. NCT03235245 is a phase II study evaluating the effect of combination targeted therapy (encorafenib and binimetinib) followed by combination immunotherapy (ipilimumab and nivolumab) compared with combination immunotherapy alone (ipilimumab and nivolumab) in patients with BRAF V600-mutated unresectable or metastatic melanoma [38]. NCT02967692 is a phase III study evaluating the safety and efficacy of the combination spartalizumab (anti-PD-1 antibody), dabrafenib (BRAF inhibitor), and trametinib (MEK inhibitor) in patients with unresectable or metastatic BRAF V600-mutated melanoma [38]. Adverse events of any grade included pyrexia (*n* = 14, 61%), arthralgia, fatigue, rash, and vomiting (*n* = 4 each, 17%) [38]. NCT02902042 is a phase I/II study investigating the efficacy and safety of encorafenib (BRAF inhibitor) combined with binimetinib (MEK inhibitor) and pembrolizumab (anti-PD-1 antibody) in patients with BRAF V600-mutated unresectable or metastatic melanoma [38]. NCT02858921 is a phase II trial designed to determine the optimal combination of drugs (dabrafenib, trametinib, and/or pembrolizumab) to reduce tumor size prior to surgery for patients with BRAF V600-mutated, resectable stage IIIB/C melanoma [38]. That study is also examining drug combinations to prevent the recurrence of melanoma after resection. 

### 4.2. Non-Small-Cell Lung Cancer (NSCLC)

Lung cancer occurs at a high incidence and is associated with mortality in both males and females. Patients with early-stage (stage I, II, and IIIA) resectable NSCLC generally undergo surgery to remove the tumor [48]. However, about 80% of lung cancers are diagnosed as advanced-stage NSCLC, for which surgical resection is not a suitable strategy. In addition, tumor recurrence can occur within a few years of surgery [49]. Therefore, chemotherapy is used the first-line treatment for patients with late-stage NSCLC patients [48]. 

Increased knowledge on the correlation between the immune system and lung cancer has led to the development of immunotherapies for the treatment of patients with NSCLC. The success of anti-PD-1/PD-L1 antibodies has been reported in multiple clinical trials, with significant responses and low toxicities observed in patients with NSCLC [50]. Chemotherapy has been shown to have minimal effects in patients with high PD-L1 levels and low expression of causative mutations. Chemotherapy was found to enhance the amount of PD-L1 on tumor cells, as well as the number of TILs [51]; hence, immune checkpoint inhibitors combined with chemotherapy have shown promising clinical results [52,53]. 

Over the past decades, there has been substantial progress in the treatment of patients with NSCLC due to the development of therapies targeting mutations in the epidermal growth factor receptor (EGFR) and anaplastic lymphoma kinase (ALK) translocations [54]. Most EGFR mutations comprise exon 19 deletions and exon 21 L858R mutations; these lead to the constitutive activation of downstream signaling events, including MAPK, phosphoinositide 3-kinase (PI3K), and signal transducer and activator of transcription (STAT) [55].

Ongoing clinical trials in NSCLC combining MAPK inhibitors and immune checkpoint inhibitors are reviewed in Table 6 [38]. NCT03991819 is a phase I/Ib study investigating the efficacy, safety, and best dose of binimetinib (MEK inhibitor) in combination with pembrolizumab (anti-PD-1 antibody) for the treatment of patients with advanced NSCLC [38]. The phase I part of study NCT03991819 comprises a dose de-escalation to determine the maximum dose of binimetinib that can be administered with pembrolizumab without having significant adverse events [38]. The efficacy, safety, tolerability, and antitumor activity of the dose determined in the phase I part will be evaluated in the phase Ib part [38]. NCT03600701 is a phase II trial assessing the effect of atezolizumab (anti-PD-L1 antibody) and cobimetinib (MEK inhibitor) for the treatment of patients with metastatic, recurrent, or refractory NSCLC [38]. NCT03581487 is a phase I/II trial analyzing the best dose of selumetinib (MEK inhibitor) in combination with durvalumab (anti-PD-L1 antibody) and tremelimumab (CTLA-4-Ab) for the treatment of patients with stage IV or recurrent NSCLC [38]. NCT03299088 is a phase Ib study investigating the safety of pembrolizumab (anti-PD-1 antibody) and trametinib (MEK inhibitor) for the treatment of patients with metastatic KRAS-mutated NSCLC [38]. NCT03225664 is a phase Ib/II trial assessing the safety and best dose of trametinib (MEK inhibitor) in combination with pembrolizumab (anti-PD-1 antibody) for the treatment of patients with metastatic, recurrent, locally advanced, and unresectable NSCLC [38]. 

### 4.3. Colorectal Cancer (CRC)

CRC is the third most common cancer worldwide [56], and OS for patients with metastatic CRC remains low. Standard treatment of CRC includes surgery followed by adjuvant chemotherapy [57,58]. Common mutations of CRC, including KRAS, p53, SMAD4, and BRAF play significant roles in CRC metastasis [57]. Clinical trials investigating MAPK inhibitors in patients with CRC are summarized in Table 7 [38]. In trial NCT01436656, a 300 mg dosage of encorafenib once daily was declared the recommended phase II dose (RP2D) [59]. The most common adverse events were nausea, myalgia, and palmar–plantar erythrodysesthesia [59]. In BRAF inhibitor-naive patients, the ORR and median PFS (mPFS) were 60% and 12.4 months, respectively [59]. In BRAF inhibitor-pretreated patients, the ORR and mPFS were 22% and 1.9 months, respectively [59]. In trial NCT00959127, the maximum tolerated dose (MTD) was 60 mg twice daily with a subsequent decrease to 45 mg twice daily due to the frequency of treatment-related ocular toxicities [60]. Common adverse events were mostly grade 1/2 rash, nausea, vomiting, diarrhea, peripheral edema, and fatigue [60]. Target inhibition was observed in serum and skin biopsy samples [60]. 

Although immune checkpoint inhibitors, including anti-PD-1 antibodies (nivolumab, pembrolizumab), anti-PD-L1 antibodies (atezolizumab, durvalumab, avelumab), and anti-CTLA-4 antibodies (ipilimumab, tremelimumab), have demonstrated efficacy in many cancers, no favorable clinical responses have been reported in patients with CRC [58]. However, immune checkpoint inhibitors may have a more favorable effect when used in combination with MAPK pathway-targeted therapy. Due to the low efficacy observed with immune checkpoint inhibitors in patients with CRC, combination strategies including targeted therapies and chemotherapy have been developed to enhance the immune response of these patients. Ongoing clinical trials investigating the combination treatment of CRC with an immune checkpoint inhibitor and MAPK-targeted therapy are presented in Table 8 [38]. NCT04044430 is a phase I/II trial investigating the effects of encorafenib (BRAF inhibitor), binimetinib (MEK inhibitor), and nivolumab (anti-PD-1 antibody) in patients with microsatellite stable (MSS), BRAF V600E-mutated metastatic CRC [38]. Trial NCT03428126 is investigating the efficacy and safety of durvalumab (anti-PD-L1 antibody) and trametinib (MEK inhibitor) in patients with MSS CRC. Durvalumab is an FDA-approved antibody used for the treatment of patients with previously treated advanced bladder cancer. Trametinib is an FDA-approved MEK inhibitor targeting advanced melanoma bearing BRAF V600E or V600K mutation with dabrafenib (BRAF inhibitor) [38]. NCT03374254 is a phase Ib study examining the efficacy and safety of combined pembrolizumab (anti-PD-1 antibody) and binimetinib (MEK inhibitor), compared with combined pembrolizumab and chemotherapy, with or without binimetinib, in patients with metastatic CRC [38]. NCT03374254 is a multi-cohort study aiming to establish the recommended phase 2 dose (RP2D) of the following five combinations (cohorts): cohort A, pembrolizumab and binimetinib; cohort B, pembrolizumab and mFOLFOX7 (oxaliplatin 85 mg/m^2^, leucovorin (calcium folinate) 400 mg/m^2^, fluorouracil (5-FU) 2400 mg/m^2^); cohort C, pembrolizumab with mFOLFOX7 and binimetinib; cohort D, pembrolizumab, FOLFIRI (irinotecan 180 mg/m^2^, leucovorin (calcium folinate) 400 mg/m^2^, 5-FU 2400 mg/m^2^ over 46–48 h); and cohort E, pembrolizumab plus FOLFIRI and binimetinib [38].

### 4.4. Pancreatic Cancer

Pancreatic ductal adenocarcinoma (PDAC) is one of the most lethal cancers worldwide, and the mortality rate is almost equivalent to the occurrence rate [61]. At the time of diagnosis, it is usually too late for surgical resection, since most patients already have advanced and metastatic disease. The 5-year survival rate for patients with PDAC is less than 10%, primarily due to the delayed diagnosis [61]. The driver mutations of PDAC include high rate of activated *KRAS* mutations, and inactivated form of mutations of *TP53, SMAD4/DPC4*, and *P16/CDKN2A* [62]. Other characteristics of PDAC include a lack of specific markers, a low response to chemotherapy, and a very low response rate to targeted therapies against MEK, PI3K, and EGFR [63,64,65]. This is related to the innate or acquired resistance of pancreatic cancer cells to these therapies [66,67]. 

The tumor microenvironment (TME) of PDAC includes a highly complex structure, in which a large proportion of cancer cells and the extracellular matrix is resistant to permeation by chemotherapeutic drugs [68]. The lack of successful clinical results in patients with PDAC is possibly due to immune suppression and escape [69]. While immune checkpoint inhibitors have demonstrated significant clinical benefits in various types of cancers, including melanoma, lung, renal, and hematological cancers [70,71,72,73,74], their efficacy in patients with PDAC is poor, with no clinical benefits observed [75].

Despite this resistance of PDAC to several therapeutic strategies, a phase Ib/II clinical trial (NCT03193190) investigating combination therapy with cobimetinib (MEK inhibitor) and atezolizumab (anti-PD-L1 antibody) in patients with metastatic PDAC is now underway; however, the results are still pending (Table 9) [38]. This study includes two patient cohorts: cohort 1, patients without any prior systemic therapy; and cohort 2, patients with one line of prior systemic therapy [38]. Another clinical trial (NCT03637491) investigating avelumab (anti-PD-L1 antibody) combined with binimetinib (MEK inhibitor) in patients with PDAC is also underway (Table 9) [38]. NCT03637491 is a phase 1b/2 study testing the effects of avelumab (anti-PD-L1 antibody), binimetinib (MEK inhibitor), and talazoparib (poly ADP ribose polymerase (PARP) inhibitor) in combination as two or three drugs for patients with metastatic PDAC and locally advanced or metastatic KRAS- or NRAS-mutated solid tumors [38]. The phase Ib part will evaluate the optimal combination of drugs, and their doses, and phase II will study the safety and efficacy of these drugs in terms of side effects and tumor growth [38].

### 4.5. Thyroid Cancer

Thyroid cancer is the most common endocrine tumors [76]. Standard therapy for patients with thyroid cancer includes surgery and treatment with radioactive iodine [77,78]. The major driver mutations of thyroid cancer include *BRAF* and *RAS* mutations, and transfusion/papillary thyroid carcinoma (*RET/PTC)* rearrangements [79]. 

Although use of a targeted therapy against the MAPK pathway generated a favorable clinical response in patients with thyroid cancer, the OS rate remains controversial [80]. This may be due to the toxicity of the MAPK inhibitor and the resistance of the tumor microenvironment. Therefore, new strategies are required to overcome these limitations. 

Reports have shown that combination therapy with a MAPK pathway-targeted inhibitor and immunotherapy may induce favorable responses in patients with thyroid cancer based on the immunomodulatory effect of MAPK inhibitors. NCT04061980 is a phase II clinical trial combining a MAPK inhibitor and a PD-1 inhibitor [38]; this study aims to assess the efficacy and safety of encorafenib (BRAF inhibitor) and binimetinib (MEK inhibitor) with or without nivolumab (anti-PD-1 antibody) in patients with BRAF V600-mutated metastatic and refractory thyroid cancer not responsive to radioiodine treatment (Table 10) [38]. Encorafenib and binimetinib have been reported to inhibit the tumor growth, and nivolumab is an anti-PD1 monoclonal antibody that boosts the immune system to suppress tumor metastasis and growth [38]. Clinical trials investigating treatments for various forms of cancer have demonstrated the feasibility of combination therapy using MAPK inhibitors and checkpoint blockers, and the ability of this approach to overcome the limitations associated with the respective monotherapy. 

## 5. Conclusions

Despite the clinical success of cancer immunotherapies, many hurdles and challenges remain. Although targeted therapy is a promising therapeutic option for various types of cancer, the development of acquired resistance remains a significant limitation. Conversely, immunotherapy has been shown to generate long-term effects, but has a low response rate. Increasingly, reports have shown that the effects of targeted therapies are enhanced when used in combination with immune cells and their microenvironments. 

Combination therapies target signal transduction cascades required for tumor cell survival and maintenance. While small-molecule inhibitors block intracellular targets, checkpoint inhibitors restore a patient’s immune system, enabling the killing of tumor cells as well as metastatic cells and those with acquired mutations. Therefore, it is important to understand the patient’s individual tumor mutation burden to select the optimal personalized combination regimen. When discerning a patient’s mutation burden, combined treatment with an immune checkpoint inhibitor and a MAPK inhibitor is an effective strategy to overcome resistance to targeted therapies. Further in-depth analyses of the mechanisms of acquired resistance are required to enable the identification of alternatives that restore tumor-specific T cells and reprogram an immunosuppressive tumor microenvironment. 

## Figures and Tables

**Table 1:** Genetic drivers of cancer.

Type of Cancer	Mutations
Melanoma	*IDH1, RB1, DDX3X, NF1, BRAF, RAS,*
Non-small-cell lung cancer	*PI3K, FGFR, DDR2, PTEN, KRAS, EGFR, BRAF, ALK*
Colorectal cancer	*APC, KRAS, TP53, SMAD4, FBXW7, BRAF, PI3K*
Pancreatic cancer	*KRAS,BRAF,TP53, CDKN2A*, *SMAD4*, *MLL3, TGFBR2, ARID1A*, *SF3B1*
Thyroid cancer	*RAS, BRAF, TP53, PI3K, RET/PTC*

**Table 2:** Food and Drug Administration (FDA)-approved targeted therapies for cancer.

Name of Drug	Company	Target	Conditions
Everolimus (Afinitor)	Novartis	mTOR	Pancreatic, gastrointestinal, or lung origin neuroendocrine tumors Renal cell carcinoma Nonresectable subependymal giant cell astrocytoma associated with tuberous sclerosis Breast cancer (HR+, HER2−)
Tamoxifen (Nolvadex)	AstraZeneca	Estrogen receptor (ER)-positive breast cancer	Breast cancer Ductal carcinoma in situ
Lapatinib (Tykerb)	GlaxoSmithKline	HER2 (ERBB2/neu), EGFR (HER1/ERBB1)	Breast cancer (HER2+)
Neratinib (Nerlynx)	Puma Biotech	HER2 (ERBB2/neu)	Breast cancer (HER2 overexpressed/amplified)
Palbociclib (Ibrance)	Pfizer	CDK4, CDK6	Breast cancer (HR+, HER2−)
Ribociclib (Kisqali)	Novartis	CDK4, CDK6	Breast cancer (HR+, HER2−)
Ado-trastuzumab emtansine (Kadcyla)	Genentech	HER2 (ERBB2/neu)	Breast cancer (HER2+)
Trastuzumab (Herceptin)	Genentech	HER2 (ERBB2/neu)	Breast cancer (HER2+) Gastric cancer (HER2+)
Erdafitinib (Balversa™)	Astex Pharmaceuticals and Janssen Pharmaceutical	FGFR	Urothelial carcinoma
Axitinib (Inlyta)	Chiron	KIT, PDGFRβ, VEGFR1/2/3	Renal cell carcinoma
Lenvatinib (Lenvima)	Eisai	VEGFR2	Renal cell carcinoma Thyroid cancer
Sorafenib (Nexavar)	Bayer	VEGFR, PDGFR, KIT, RAF	Hepatocellular carcinoma Renal cell carcinoma Thyroid carcinoma
Temsirolimus (Torisel)	Pfizer	mTOR	Renal cell carcinoma
Pazopanib (Votrient)	GlaxoSmithKline	VEGFR, PDGFR, KIT	Renal cell carcinoma
Cabozantinib (Cabometyx (tablet), Cometriq (capsule]))	Exelixis	FLT3, KIT, MET, RET, VEGFR2	Medullary thyroid cancer Renal cell carcinoma
Afatinib (Gilotrif)	Boehringer Ingelheim	EGFR (HER1/ERBB1), HER2 (ERBB2/neu)	Non-small-cell lung cancer (with EGFR exon 19 deletions or exon 21 substitution (L858R) mutations)
Alectinib (Alecensa)	Genentech	ALK	Non-small-cell lung cancer (with ALK fusion)
Brigatinib (Alunbrig)	Takeda Pharmaceutical	ALK	Non-small-cell lung cancer (ALK+)
Ceritinib (Zykadia)	Novartis	ALK	Non-small-cell lung cancer (with ALK fusion)
Crizotinib (Xalkori)	Pfizer	ALK, MET, ROS1	Non-small-cell lung cancer (with ALK fusion or ROS1 gene alteration)
Erlotinib (Tarceva)	Roche	EGFR (HER1/ERBB1)	Non-small-cell lung cancer (with EGFR exon 19 deletions or exon 21 substitution (L858R) mutations) Pancreatic cancer
Gefitinib (Iressa)	AstraZeneca	EGFR (HER1/ERBB1)	Non-small-cell lung cancer (with EGFR exon 19 deletions or exon 21 substitution (L858R) mutations)
Osimertinib (Tagrisso)	AstraZeneca	EGFR	Non-small-cell lung cancer (with EGFR T790M mutation)
Cobimetinib (Cotellic)	Genentech	MEK	Melanoma (with BRAF V600E or V600K mutation)
Dabrafenib (Tafinlar)	GlaxoSmithKline	BRAF	Melanoma (with BRAF V600 mutation) Non-small-cell lung cancer (with BRAF V600E mutation)
Necitumumab (Portrazza)	Eli Lilly	EGFR (HER1/ERBB1)	Squamous non-small-cell lung cancer
Bortezomib (Velcade)	Takeda	Proteasome	Multiple myeloma Mantle cell lymphoma
Bosutinib (Bosulif)	Pfizer	ABL	Chronic myelogenous leukemia (Philadelphia chromosome positive)
Carfilzomib (Kyprolis)	Onyx	Proteasome	Multiple myeloma
Dasatinib (Sprycel)	Bristol-Myers Squibb	ABL	Chronic myelogenous leukemia (Philadelphia chromosome positive) Acute lymphoblastic leukemia (Philadelphia chromosome positive)
Enasidenib (Idhifa)	Agios Pharmaceuticals/Celgene	IDH2	Acute myeloid leukemia (with IDH2 mutation)
Venetoclax (Venclexta)	AbbVie and Roche	BCL2	Chronic lymphocytic leukemia (with 17p deletion)
Ibrutinib (Imbruvica)	Johnson &Johnson	BTK	Mantle cell lymphoma Chronic lymphocytic leukemia Waldenstrom’s macroglobulinemia
Idelalisib (Zydelig)	Gilead	PI3Kδ	Chronic lymphocytic leukemia Follicular B-cell non-Hodgkin’s lymphoma Small lymphocytic lymphoma
Ixazomib (Ninlaro)	Takeda Pharmaceutical	Proteasome	Multiple myeloma
Midostaurin (Rydapt)	Novartis	FLT3	Acute myeloid leukemia (FLT3+)
Nilotinib (Tasigna)	Novartis	ABL	Chronic myelogenous leukemia (Philadelphia chromosome positive)
Ponatinib (Iclusig)	ARIAD	ABL, FGFR1–3, FLT3, VEGFR2	Chronic myelogenous leukemia Acute lymphoblastic leukemia (Philadelphia chromosome positive)
Trametinib (Mekinist)	GlaxoSmithKline	MEK	Melanoma (with BRAF V600 mutation) Non-small-cell lung cancer (with BRAF V600E mutation)
Vemurafenib (Zelboraf)	Genentech	BRAF	Melanoma (with BRAF V600 mutation)
Cetuximab (Erbitux)	Eli Lilly	EGFR (HER1/ERBB1)	Colorectal cancer (KRAS wild type) Squamous cell cancer of the head and neck
Ziv-aflibercept (Zaltrap)	Sanofi-Aventis	PIGF, VEGFA/B	Colorectal cancer
Panitumumab (Vectibix)	Amgen	EGFR (HER1/ERBB1)	Colorectal cancer (KRAS wild type)
Ramucirumab (Cyramza)	Eli Lilly	VEGFR2	Colorectal cancer Gastric cancer or gastroesophageal junction (GEJ) adenocarcinoma Non-small-cell lung cancer
Regorafenib (Stivarga)	Bayer	KIT, PDGFRβ, RAF, RET, VEGFR1–3	Colorectal cancer Gastrointestinal stromal tumors Hepatocellular carcinoma
Rucaparib (Rubraca)	Clovis Oncology	PARP	Ovarian cancer (with BRCA mutation)
Niraparib (Zejula)	Tesaro	PARP	Ovarian cancer Fallopian tube cancer Peritoneal cancer
Olaparib (Lynparza)	AstraZeneca	PARP	Ovarian cancer (with BRCA mutation)
Denosumab (Xgeva)	Amgen	RANKL	Giant cell tumor of the bone
Dinutuximab (Unituxin)	United Therapeutics	B4GALNT1 (GD2)	Pediatric neuroblastoma
Imatinib (Gleevec)	Novartis	KIT, PDGFR, ABL	GI stromal tumor (KIT+) Dermatofibrosarcoma protuberans Multiple hematological malignancies including Philadelphia chromosome-positive ALL and CML
Sonidegib (Odomzo)	Novartis	Smoothened	Basal cell carcinoma
Vismodegib (Erivedge)	Roche	PTCH, Smoothened	Basal cell carcinoma
Olaratumab (Lartruvo)	Eli Lilly	PDGFRα	Soft tissue sarcoma
Ruxolitinib (Jakafi)	Incyte	JAK1/2	Myelofibrosis
Tofacitinib (Xeljanz)	Pfizer	JAK3	Rheumatoid arthritis
Vandetanib (Caprelsa)	AstraZeneca	EGFR (HER1/ERBB1), RET, VEGFR2	Medullary thyroid cancer

**Table 3:** FDA-approved drugs for immunotherapy.

Name of Drug	Company	Target	Conditions
Alemtuzumab (Campath)	Sanofi	CD52	B-cell chronic lymphocytic leukemia
Atezolizumab (Tecentriq)	Genentech	PD-L1	Urothelial carcinoma Non-small-cell lung cancer
Avelumab (Bavencio)	Merck KGaA and Pfizer	PD-L1	Merkel cell carcinoma Urothelial cancer
Blinatumomab (Blincyto)	Amgen	CD19/CD3	Acute lymphoblastic leukemia (precursor B-cell)
Brentuximab vedotin (Adcetris)	Takeda Pharmaceutical	CD30	Hodgkin lymphoma Anaplastic large cell lymphoma
Canakinumab (Ilaris)	Novartis	IL-1β	Juvenile idiopathic arthritis Cryopyrin-associated periodic syndromes
Daratumumab (Darzalex)	Janssen Pharmaceutical	CD38	Multiple myeloma
Durvalumab (Imfinzi)	MedImmune/AstraZeneca	PD-L1	Urothelial carcinoma Non-small-cell lung cancer
Elotuzumab (Empliciti)	Bristol-Myers Squibb	SLAMF7 (CS1/CD319/CRACC)	Multiple myeloma
Ibritumomab tiuxetan (Zevalin)	Biogen IDEC	CD20	Non-Hodgkin’s lymphoma
Ipilimumab (Yervoy)	Bristol-Myers Squibb	CTLA-4	Melanoma Renal cell carcinoma
Nivolumab (Opdivo)	Bristol-Myers Squibb	PD-1	Colorectal cancer (dMMR and MSI-H) Head and neck squamous cell carcinoma Hepatocellular carcinoma Hodgkin lymphoma Melanoma Non-small-cell lung cancer Renal cell carcinoma Urothelial carcinoma
Obinutuzumab (Gazyva)	Roche	CD20	Chronic lymphocytic leukemia Follicular lymphoma
Ofatumumab (Arzerra, HuMax-CD20)	Roche	CD20	Chronic lymphocytic leukemia
Pembrolizumab (Keytruda)	Merck &Co	PD-1	Classical Hodgkin lymphoma Colorectal cancer (MSI-H/dMMR) Gastric cancer Melanoma Non-small-cell lung cancer (PD-L1+) Head and neck squamous cell carcinoma Urothelial cancer Solid tumors (MSI-H/dMMR)
Rituximab (Rituxan, Mabthera)	Roche	CD20	Non-Hodgkin’s lymphoma Chronic lymphocytic leukemia Rheumatoid arthritis Granulomatosis with polyangiitis
Rituximab/hyaluronidase human (Rituxan Hycela)	Roche	CD20	Chronic lymphocytic leukemia Diffuse large B-cell lymphoma Follicular lymphoma
Siltuximab (Sylvant)	Janssen Pharmaceutical	IL-6	Multicentric Castleman’s disease
Tocilizumab (Actemra)	Genentech	IL-6R	Rheumatoid arthritis Juvenile idiopathic arthritis
Tositumomab (Bexxar)	Corixa	CD20	Non-Hodgkin’s lymphoma

**Table 4:** FDA-approved checkpoint inhibitors.

Name of Drug	Company	Target	Indications
Ipilimumab	Bristol-Myers Squibb	CTLA-4	Unresectable or metastatic melanoma
Tremelimumab	AstraZeneca	CTLA-4	Unresectable malignant mesothelioma Metastatic melanoma Non-small-cell lung cancer
Nivolumab	Bristol-Myers	PD-1	Unresectable or metastatic melanoma Metastatic non-small-cell lung cancer Advanced renal cell carcinoma Recurrent or metastatic head and neck squamous cell carcinoma Locally advanced or metastatic urothelial carcinoma Hepatocarcinoma
Pembrolizumab	Merck	PD-1	Unresectable or metastatic melanoma Metastatic non-squamous non-small-cell lung cancer Recurrent or metastatic head and neck squamous cell carcinoma Refractory Hodgkin lymphoma Locally advanced or metastatic urothelial carcinoma
Atezolizumab	Roche	PD-L1	Advanced or metastatic urothelial carcinoma Metastatic non-small-cell lung cancer
Avelumab	Merck Pfizer	PD-L1	Locally advanced or metastatic urothelial carcinoma
Durvalumab	MedImmune/AstraZeneca	PD-L1	Urothelial carcinoma Non-small-cell lung cancer

**Table 5:** Clinical trials of therapies targeting the MAPK pathway and immunotherapy in patients with melanoma.

National Clinical Trial (NCT) Number	Title	Status	Conditions	Interventions	Phase	Start Date
NCT 01400451	Ph I Ipilimumab Vemurafenib Combo in Patients with v-Raf Murine Sarcoma Viral Oncogene Homolog B1 (BRAF)	Terminated (unexpected grade 2/3 hepatotoxicity)	•Melanoma	•Drug: Ipilimumab (BMS-734016) •Drug: Vemurafenib	Phase 1	November 2011
NCT 01673854	Phase II Safety Study of Vemurafenib Followed by Ipilimumab in Subjects with V600 BRAF Mutated Advanced Melanoma	Completed (no severe hepatotoxicity, but reported a grade 3/4 skin adverse event)	•Melanoma	•Drug: Ipilimumab •Biological: Vemurafenib	Phase 2	13 September 2012
NCT 03554083	Neoadjuvant Combination Targeted and Immunotherapy for Patients with High-Risk Stage III Melanoma	Recruiting	•Clinical stage iii cutaneous melanoma ajcc v8 •Pathologic Stage III Cutaneous Melanoma AJCC v8 •Pathologic Stage IIIA Cutaneous Melanoma AJCC v8 •Pathologic Stage IIIB Cutaneous Melanoma AJCC v8 •Pathologic Stage IIIC Cutaneous Melanoma AJCC v8 •Pathologic Stage IIID Cutaneous Melanoma AJCC v8	•Drug: Atezolizumab •Drug: Cobimetinib •Drug: Vemurafenib	Phase 2	22 June 2018
NCT 03235245	Immunotherapy with Ipilimumab and Nivolumab Preceded or Not by a Targeted Therapy with Encorafenib and Binimetinib	Recruiting	•Unresectable Stage III Melanoma •Stage IV Melanoma	•Drug: Nivolumab + Ipilimumab •Drug: Encorafenib+ Binimetinib	Phase 2	30 October 2018
NCT 02967692	A Study of the Anti-PD1 Antibody PDR001, in Combination with Dabrafenib and Trametinib in Advanced Melanoma	Recruiting	•Melanoma	•Biological: Spartalizumab (PDR001) •Other: Placebo •Drug: Dabrafenib •Drug: Trametinib	Phase 3	17 February 2017
NCT 02902042	Encorafenib + Binimetinib + Pembrolizumab in Patients with Unresectable or Metastatic BRAF V600 Mutant Melanoma	Recruiting	•Malignant Melanoma	•Drug: Encorafenib •Drug: Binimetinib •Drug: Pembrolizumab •Drug: Pembrolizumabalone	•Phase 1 •Phase 2	24 April 2018
NCT 02858921	Neoadjuvant Dabrafenib, Trametinib and/or Pembrolizumab in BRAF Mutant Resectable Stage III Melanoma	Recruiting	•Melanoma	•Melanoma •Drug: Dabrafenib •Drug: Trametinib •Drug: Pembrolizumab	Phase 2	8 November 2017

**Table 6:** Clinical trials of therapies targeting the MAPK pathway and immunotherapies in patients with NSCLC.

National Clinical Trial (NCT) Number	Title	Status	Conditions	Interventions	Phase	Start Date
NCT 03991819	Study of Binimetinib in Combination with Pembrolizumab in Advanced Non-Small-Cell Lung Cancer	Recruiting	•Non-Small-Cell Carcinoma	•Drug: Binimetinib •Drug: Pembrolizumab	•Phase 1	20 September 2019
NCT 03600701	Atezolizumab and Cobimetinib in Treating Patients with Metastatic, Recurrent, or Refractory Non-Small-Cell Lung Cancer	Recruiting	•Recurrent Lung Non-Small-Cell Carcinoma •Refractory Lung Non-Small-Cell Carcinoma •Stage IV Lung Non- Small-Cell Cancer AJCC v7	•Drug: Atezolizumab •Drug: Cobimetinib	Phase 2	20 July 2018
NCT 03581487	Durvalumab, Tremelimumab, and Selumetinib in Treating Participants with Recurrent or Stage IV Non-Small-Cell Lung Cancer	Recruiting	•Recurrent Lung Non-Small-Cell Carcinoma •Stage IV Lung Cancer AJCC v8 •Stage IVA Lung Cancer AJCC v8 •Stage IVB Lung Cancer AJCC v8	•Biological: Durvalumab •Drug: Selumetinib •Biological:Tremelimumab	•Phase 1 •Phase 2	1 April 2019
NCT 03299088	Pembrolizumab and Trametinib in Treating Patients with Stage IV Non-Small-Cell Lung Cancer and KRAS Gene Mutations	Recruiting	•KRAS Gene Mutation •Metastatic Non- Squamous Non- Small Cell Lung Carcinoma •Recurrent Non- Squamous Non- Small Cell Lung Carcinoma •Stage IV Non-Small-Cell Lung Cancer AJCC v7	•Biological: Pembrolizumab •Drug: Trametinib	Phase 1	26 June 2018
NCT 03225664	Trametinib and Pembrolizumab in Treating Patients with Recurrent Non-Small-Cell Lung Cancer That Is Metastatic, Unresectable, or Locally Advanced	Recruiting	•Metastatic Lung Non-Small-Cell Carcinoma •Recurrent Lung Non-Small-Cell Carcinoma •Stage III Lung Cancer AJCC v8 •Stage IIIA Lung Cancer AJCC v8 •Stage IIIB Lung Cancer AJCC v8 •Stage IIIC Lung Cancer AJCC v8 •Stage IV Lung Cancer AJCC v8 •Stage IVA Lung Cancer AJCC v8 •Stage IVB Lung Cancer AJCC v8 •Unresectable Lung Non-Small-Cell Carcinoma	•Biological: Pembrolizumab •Other: Pharmacokinetic Study •Drug: Trametinib	•Phase 1 •Phase 2	3 February 2018

**Table 7:** Clinical trials of therapies targeting the MAPK pathway in patients with colorectal cancer.

National Clinical Trial (NCT) Number	Title	Status	Conditions	Interventions	Phase	Start Date
NCT 01436656	A Phase I Study of Oral LGX818 in Adult Patients with Advanced or Metastatic BRAF Mutant Melanoma	Active, not recruiting	•Melanoma and Metastatic Colorectal Cancer	•Drug: LGX818	•Phase 1	September 2011
NCT 00959127	A Study of ARRY-438162 (MEK162) in Patients with Advanced Cancer	Completed	•Advanced Solid Tumors •Advanced or Metastatic Biliary Cancer •Metastatic Colorectal Cancer	•Drug: ARRY-438162 (MEK162), MEK inhibitor; oral	Phase 1	August 2009

**Table 8:** Clinical trials investigating therapies targeting the MAPK pathway and immunotherapies in patients with colorectal cancer.

National Clinical Trial (NCT) Number	Title	Status	Conditions	Interventions	Phase	Start Date
NCT 04044430	Encorafenib, Binimetinib, and Nivolumab in Treating Patients with Microsatellite Stable BRAFV600E Metastatic Colorectal Cancer	Not yet recruiting	•Metastatic Colon Adenocarcinoma •Metastatic Colorectal Adenocarcinoma •Metastatic Microsatellite Stable Colorectal Carcinoma •Metastatic Rectal Adenocarcinoma •Stage III Colon Cancer •Stage III Colorectal Cancer •Stage III Rectal Cancer •Stage IIIA Colon Cancer •Stage IIIA Colorectal Cancer •Stage IIIA Rectal Cancer •and 18 more	•Drug: Binimetinib •Drug: Encorafenib •Biological: Nivolumab •Other: Questionnaire Administration	•Phase 1 •Phase 2	1 December 2019
NCT 03428126	Study of Durvalumab (MEDI4736) (Anti-PD-L1) and Trametinib (MEKi) in MSS Metastatic Colon Cancer	Enrolling by invitation	•Malignant Neoplasms of Digestive Organs •Colorectal Cancer •Colon Cancer	•Drug: Durvalumab •Drug: Trametinib	Phase 2	21 March 2018
NCT 03374254	Safety and Efficacy of Pembrolizumab (MK-3475) Plus Binimetinib Alone or Pembrolizumab Plus Chemotherapy with or without Binimetinib in Metastatic Colorectal Cancer (mCRC) Participants (MK-3475-651)	Recruiting	•Metastatic Colorectal Cancer	•Biological: Pembrolizumab •Drug: Binimetinib •Drug: Oxaliplatin •Drug: Leucovorin •Drug: 5-Fluorouracil [5-FU] •Drug: Irinotecan	Phase 1	16 February 2018

**Table 9:** Clinical trials of therapies targeting the MAPK pathway and immunotherapies in patients with pancreatic cancer.

National Clinical Trial (NCT) Number	Title	Status	Conditions	Interventions	Phase	Start Date
NCT 03193190	A Study of Multiple Immunotherapy-Based Treatment Combinations in Participants with Metastatic Pancreatic Ductal Adenocarcinoma (Morpheus Pancreatic Cancer)	Recruiting	•Pancreatic Adenocarcinoma	•Drug: NabPaclitaxel •Drug: Gemcitabine •Drug: Oxaliplatin •Drug: Leucovorin •Drug: Fluorouracil •Drug: Atezolizumab •Drug: Cobimetinib •Drug: PEGPH20 •Drug: BL-8040 •Drug: Selicrelumab •and 3 more	•Phase 1 •Phase 2	5 July 2017
NCT 03637491	A Study of Avelumab, Binimetinib and Talazoparib in Patients with Locally Advanced or Metastatic RAS-mutant Solid Tumors	Recruiting	•Pancreatic Cancer	•Drug: Avelumab •Drug: Binimetinib •Drug: Talazoparib	Phase 2	15 August 2018

**Table 10:** Clinical trials investigating combined MAPK pathway-targeted therapy and immunotherapy in patients with thyroid cancer.

National Clinical Trial (NCT) Number	Title	Status	Conditions	Interventions	Phase	Start Date
NCT 04061980	Encorafenib and Binimetinib with or without Nivolumab in Treating Patients with Metastatic Radioiodine Refractory BRAF V600 Mutant Thyroid Cancer	Not yet recruiting	•BRAF NP_004324.2:p.V600M •BRAF V600E Mutation Present •Metastatic Thyroid Gland Carcinoma •Refractory Thyroid Gland Carcinoma •Stage IV Differentiated Thyroid Gland Carcinoma AJCC v8 •Stage IVA Differentiated Thyroid Gland Carcinoma AJCC v8 •Stage IVB Differentiated Thyroid Gland Carcinoma AJCC v8	•Drug: Binimetinib •Drug: Encorafenib •Biological: Nivolumab	Phase 2	30 August 2019

## Abbreviations

MAPK	mitogen-activated protein kinase
NSCLC	non-small-cell lung carcinoma
MEK	MAPK/ERK kinase
PD-1	programmed death protein 1
PD-L1	programmed death-ligand 1
PD-L2	programmed death-ligand 2
CTLA-4	cytotoxic T cell associated antigen 4
US FDA	United States Food and Drug Administration
Tim-3	T-cell immunoglobulin and mucin-domain containing-3
IL-2	interleukin 2
PFS	progression-free survival
OS	overall survival
EGFR	epidermal growth factor receptor
ALK	anaplastic lymphoma kinase
PI3K	phosphoinositide 3-kinase
STAT	signal transducer and activator of transcription
CRC	colorectal cancer
mCRC	metastatic colorectal cancer
MSS	microsatellite stable
mAbs	monoclonal antibodies
PDAC	pancreatic ductal adenocarcinoma
PARP	poly ADP ribose polymerase
TME	tumor microenvironment
RET/PTC	rearranged in transfusion/papillary thyroid carcinoma
RP2D	recommended phase II dose
ORR	overall response rate
mPFS	median progression-free survival
MTD	maximum tolerated dose
